# The Action of Cohesive Foil under the Instrument

**Published:** 1873-06

**Authors:** E. L. Hunter

**Affiliations:** Enfield, N. C.


### ARTICLE II.

### *The Action of Cohesive Foil Under the Instrument.*

BY E. L. HUNTER, D. D. S., ENFIELD, N. C.

We beg the patience of our brethren in the endeavor to set forth a few principles relating to the action of cohesive gold foil under the plugget, together with a few strictures. It is designed as a reply to Dr. M. S. Dean's article in the

*Dental Cosmos* for March, 1873, headed "Large Crown Cavities with Walls Unbroken."

A free, bold exchange of opinion is often fruitful of good. It is a search for truth, and not either egotism or want of respect for the doctor's ability, which prompts us to pursue this subject. A clear view of a few mechanical principles is chiefly what is necessary, out of the light of which many an "imperceptible space" has been made. We were asked when cohesive foil under pressure, ceased to draw upon itself. That question is a very important one; our answer is, under two conditions: 1st, when from the form of pellets, it has been reduced to absolute solidity; 2nd, *when it has received its greatest pressure prior to absolute solidity*; and the latter is the condition we have most to do with. *Let each pellet be introduced with the greatest force which is ever to come upon it*, let it be accurately adapted to the walls, and it will remain so. On the contrary, introduce several pellets with moderate pressure, and then condense with a greater force, or introduce with a large point, and condense with a small one, which is nearly the same thing; of course there will be a drawing from the walls for the very same reason that a pellate draws in laterally under the first stroke of the point

Is gold foil elastic? After it has assumed somewhat the density of a plug, it is sufficiently so to effect our operations either for good or evil. If it be used to create pressure against tooth walls, to a certain extent it is good, but if used in such a manner as to rebound from them, it is evil. It was long successfully used by being set up with the wedge between parallel walls in the centre of non-cohesive fillings, being made to press the gold outward against the walls, which so far as the filling of cavities goes, is the wedge proper. But if this elastic pressure be made use of on the margin of a cohesive filling where a V shaped space has been left, in which is to be condensed "light unannealed foil," which is to be "held in by overlapping cohesive gold;" what is to become of the soft foil when the overlapping cohesive (gold) " is removed rather by the process of finishing or attrition, (the cohesive being imperfect on one side,) and the *pressure* is to bear, which is far from 'lateral' on either, but at right angles to a line of its sides?—V shaped wedges never remain where they are driven, unless there is something more than usual to hold them there. A wedge which is designed not to fly out as a result of somewhat contending pressure, would have to be very much sharper than a V: in fact a V shaped substance could hardly be called a wedge.

We think there are better methods, particularly when the work approaches completion, of filling up this elastic pressure against tooth walls; then it is made to act from a floor of perfect cohesion, if the floor is concave, (*slightly*) the pressure may be small; if level, it may be greater, that is so far as a condition prior to absolute solidity is concerned. In this condition there may be nice adaptation to the walls, and also ample solidity of the gold *without great* pressure, whereas when a wedge is used next to walls, if there is solidity, there must be great pressure, which might not be very desirable. When there is either a level or slightly concave surface, one has almost absolute power over lateral pressure with the privilege of solidity. If the gold is both plastic and cohesive, the most accurate adaptations may be made to the walls. I suppose a cavity nearly full—place one end of a section of wall $\frac{1}{4}$ inch long on the centre of the work, take the smallest point you intend using (Voney's wax is small enough ordinarily,) and condense every part of it, which is over the filling, commencing at the centre and ending at the periphery; there will then be a part of the pellet standing up by the wall, turn the end of this down on the work and condense as before, then drive the remainder down and directly by and touching the wall. If more elastic pressure is wanted, do the malleting on this slightly elevated peripheral ring with a smaller point. If you increase the length of this ring, it will not matter practically, unless you carry it on quite out of reason. Its increase in length is almost entirely produced by a slight swell outward, and even with the point where the blow is struck. Where gold foil is manipulated in this manner, we have never seen even a stain to show that there was not accurate adaptation. If one is compelled to use gold which is annealed to hardness, against thin walls, we know no method not attended with great uncertainty. To this condition of harshness is due many failures, it cannot be accurately adapted to the walls without more force than it is always judicious to apply, unless the pellets are so small as to make the operation too tedious. We think if the gold beaters would give us some unvarying rule by which we can re-establish the cohesiveness of thin gold when it has been lost, without destroying its plasticity, that they would greatly benefit us. The methods among dentists generally, are somewhat slow, and attended with some uncertainty. We could suggest some arrangement with a thermometer.

Improper annealing and untimely condensation has been our stumbling stone, and observation compels us to think it has been the same thing with many others, even including Dr. Dean, who thinks that "margins" can be made perfect only by the principle of wedging.

---

